# Guideline Compliance of Artificial Intelligence–Generated Diet Plans After Bariatric Surgery: A Cross-Sectional Simulation Comparing ChatGPT-4o, DeepSeek and Grok-3

**DOI:** 10.3390/nu17243957

**Published:** 2025-12-18

**Authors:** Aylin Bolat Yilmaz, Emre Batuhan Kenger, Tugce Ozlu Karahan, Duygu Saglam, Murat Bas

**Affiliations:** 1 Department of Nutrition and Dietetics, Institute of Health Sciences, Acibadem Mehmet Ali Aydinlar University, 34752 Istanbul, Türkiye; aylinbolattt@gmail.com; 2 Department of Nutrition and Dietetics, Faculty of Health Sciences, Istanbul Bilgi University, 34440 Istanbul, Türkiye; emre.kenger@bilgi.edu.tr (E.B.K.); tugce.karahan@bilgi.edu.tr (T.O.K.); 3 Department of Nutrition and Dietetics, Faculty of Health Sciences, Acibadem Mehmet Ali Aydinlar University, 34752 Istanbul, Türkiye

**Keywords:** sleeve gastrectomy, postoperative nutrition, large language models

## Abstract

**Background/Objectives**: Artificial intelligence (AI)-based tools are increasingly being used in tailored nutrition management, and evaluating their compliance with guidelines is significant in clinically sensitive areas, including bariatric surgery. This study aimed to investigate the extent to which diet plans recommended by AI models in the early period following sleeve gastrectomy (SG) align with current clinical nutrition guidelines (ASMBS, AACE/TOS). **Methods**: A total of 360 menu plans were generated using three AI platforms—ChatGPT-4o, DeepSeek V3, and Grok-3—for 40 simulated patients (20 females, 20 males; BMI 32–45 kg/m^2^) across three postoperative stages: liquid (day 5), puree (day 16), and solid (day 35). The energy and nutrient contents of the menus were analyzed using BeBiS 8.1; an experienced dietitian assessed compliance with the guidelines using a structured checklist. Nutrient intakes and guideline compliance scores were examined using within-patient Friedman tests followed by Bonferroni-adjusted pairwise comparisons. **Results**: ChatGPT-4o demonstrated the highest overall compliance scores, particularly in the liquid and puréed phases, while DeepSeek produced higher values for several micronutrients. All models showed substantial gaps in essential postoperative recommendations, most notably thiamine and multivitamin supplementation. **Conclusions**: Although LLMs can generate partially guideline-concordant postoperative diet plans, they consistently omit several critical elements of bariatric nutrition care. These findings indicate that LLM-generated menus may serve as supportive educational tools, and diet planning must be performed under the guidance of a specialist dietitian. This simulation does not assess clinical safety, efficacy, or patient outcomes and should not be used as a substitute for dietitian-led postoperative nutrition care.

## 1. Introduction

Obesity is a chronic and complex disease characterized by excessive fat accumulation in the body that can negatively affect health [1]. Obesity elevates the risk of type 2 diabetes, heart disease, certain types of cancer, bone-related issues, and impaired reproductive function, as well as reduces the quality of life by affecting sleep patterns and mobility [2]. Metabolic and bariatric surgery (MBS) is a method employed in managing obesity; it provides up to 60% excess weight loss (EWL) at 3–5 years postoperatively [3]. Procedures such as Roux-en-Y gastric bypass and sleeve gastrectomy alter gastrointestinal anatomy, promoting weight loss through restriction, malabsorption, and hormonal modulation (e.g., increased GLP-1 secretion) [2]. Weight loss after MBS can vary widely depending on several factors, including the patient’s preoperative metabolic profile, adherence to postoperative dietary and physical activity recommendations, behavioral changes, and overall lifestyle factors [3,4]. The success of MBS relies not only on the surgical intervention, but also on a thorough understanding of the changes in eating behavior that occur pre- and postoperatively [4]

Recently, artificial intelligence (AI) technologies have rapidly advanced in healthcare, reaching significant potential in providing targeted solutions and interventions, and have begun to be increasingly used in all stages of the nutrition care process [5]. Integrating AI into nutrition science is rapidly increasing, and this field provides significant opportunities in terms of tailored health management, more accurate nutritional assessments, and disease prevention strategies [5,6]. In particular, ChatGPT, developed by OpenAI in 2022, could be an effective tool in nutrition counseling owing to its user-friendliness, speed, and comprehensive data access [7,8]. Moreover, ChatGPT has demonstrated the potential to provide personalized nutrition recommendations for type 2 diabetes, obesity, metabolic dysfunction-associated steatotic liver disease, and cardiovascular diseases [7,9]. Although large language model (LLM)-based tools, including DeepSeek AI and Grok, have been recently developed alongside ChatGPT, data on the reliability of these tools in the field of health, and particularly nutrition, are relatively scarce [10].

The potential of AI to mitigate the risk of complications and accelerate recovery by providing support in various areas, including assessment, decision-making, and individualized management during the MBS process, has been noted in the literature [11,12]. However, studies evaluating AI-based dietary interventions following MBS are relatively limited; to our knowledge, no studies have compared and evaluated the compliance of nutrition programs developed by different LLMs following MBS with guidelines. To address this knowledge gap, we designed a simulation study to critically evaluate the effectiveness and appropriateness of diet programs developed by ChatGPT, DeepSeek AI, and Grok for patients who underwent MBS, specifically for SG. The primary objective was to compare the model’s total guideline compliance scores across postoperative phases. Furthermore, the study included exploratory analyses of the generated menus to evaluate potential differences in their energy and macro- and micronutrient contents, as well as the frequency of individual guideline recommendations. This study does not involve real patients, postoperative outcomes, or any evaluation of nutritional safety in clinical practice, and it should not be regarded as a substitute for dietitian-led postoperative care. Instead, the analysis represents a fully synthetic, simulation-based stress test designed to observe how leading AI-based large language models respond to a single, standardized prompt within the context of MBS nutrition. All patient profiles were simulated, and the postoperative diet plans were generated by three advanced large language models—ChatGPT-4o, Grok-3, and DeepSeek—selected for their increasing relevance in digital health applications and their differing algorithmic approaches to producing nutrition-related content. Based on earlier studies using AI in nutrition and health [9,13], we expect that the three AI platforms will show different results in nutrient content and guideline compliance.

## 2. Materials and Methods

### 2.1. Patients Simulation

In our study, the performance of ChatGPT-4o, Grok-3, and DeepSeek V3 in generating postoperative nutrition plans was evaluated using 40 independent example patient profiles created by the researchers, a relevant approach given that SG has become the most frequently performed bariatric procedure worldwide over the past two decades [2]. These profiles served as standardized prompt scenarios designed to test AI responses across a range of body weights, ages, and sexes commonly encountered in clinical bariatric practice. To ensure consistency and comparability between AI outputs, researchers manually constructed 40 simulated patients (20 male, 20 female; BMI range 32–45 kg/m^2^; age 19–62 years). All profiles were assigned a sedentary activity level to avoid variability due to differing energy expenditure assumptions. Each AI model received the same set of 40 prompts for postoperative days 5, 16, and 35. For each scenario, the AI-generated one-day dietary plan was extracted and analyzed. No iterative prompting, optimization commands, or corrective feedback was provided after the initial prompt to maintain methodological neutrality. This study did not involve human participants, patient records, or clinical interventions. All patient profiles were fully simulated, and all data were AI-generated; therefore, ethical approval and informed consent were not required.

### 2.2. Prompts Entered into AI Models

The prompts entered into the AI models specify the individual’s age, height, body weight, and the number of days since SG. No specific weight loss goal is specified for individuals. The language and sentence structure of the prompts are uncomplicated for individuals to consult a healthcare professional, rather than providing guidance that could be useful for professional advice, and have been entered only once into all models. To improve the planned diet, no additional commands were added following the prompt entry. To ensure no bias in this study, the free versions of the ChatGPT-4o, Grok-3, and DeepSeek V3 models were employed. The single-day menu plans generated by AI models for each simulated patient were analyzed according to the postoperative nutrition periods. The following are the prompt examples used in this study:

“Can you prepare a nutrition plan for a 24-year-old male who is 169 cm tall, weighs 115 kg, is sedentary, and is on day 35 post-sleeve gastrectomy?”

“Can you prepare a nutrition plan for a 21-year-old female who is 170 cm tall, weighs 120 kg, is sedentary, and is on day 16 post-sleeve gastrectomy?”

All large language models were accessed through their official web interfaces between 1 and 15 March 2025 using default system settings. The models used were ChatGPT-4o (OpenAI), Grok-3 (xAI), and DeepSeek-R1/Chat (DeepSeek). Because these interfaces do not allow users to modify sampling parameters such as temperature, all models operated with their built-in stochastic (random) generation settings. Accordingly, the menu plans produced in this study reflect each model’s default stochastic sampling behavior.

To establish a controlled zero-shot evaluation environment, the prompts were intentionally restricted to age, gender, height, body weight, sedentary status, and postoperative day. This study was not designed to replicate individualized clinical decision-making; rather, it serves as a stress test that examines standardized model behavior under minimal-input conditions.

As a sensitivity analysis, a subset of three simulated patients was selected, and their prompts were rerun across all three diet phases and all three models at two additional time points. The first prompt submission occurred nine months earlier, while the second and third submissions were generated on the same day after clearing the interface to ensure independent outputs. This approach allowed us to examine the stability of AI-generated results by assessing how much the menus and their corresponding guideline-compliance scores varied across independent generations of the same inputs.

### 2.3. Diet Plan Evaluation

The content and suitability of diet plans developed for simulated patients who underwent SG using AI-based systems (ChatGPT-4o, Grok-3, and DeepSeek) in terms of post-metabolic and bariatric surgery nutrition were evaluated on the basis of the joint guidelines of the American Society for Metabolic and Bariatric Surgery (ASMBS) and the American Association of Clinical Endocrinologists/the Obesity Society (AACE/TOS/ASMBS). Nutritional recommendations according to the postoperative periods outlined in these joint guidelines are summarized in Table 1.

The compliance of AI-generated diet plans with post- metabolic and bariatric surgery nutrition guidelines was assessed using a structured checklist derived from ASMBS, AACE/TOS/ASMBS [14]. The checklist includes essential items specific to the relevant periods, such as protein targets, multivitamin use, fluid intake, fluid–solid separation, thiamine and iron supplementation, and suitability for a liquid/soft diet. To ensure methodological transparency, clear definitions were created for each checklist item. The minimum content and expression criteria required for an item to be coded as ‘present’ were determined, and each item was scored on a binary scale (1 = present, 0 = absent). The coding criteria for each item are presented in Table 2. An expert dietitian (A.B.Y.) with experience in metabolic and bariatric surgery and clinical nutrition conducted the evaluation process; the extent to which each plan and the recommendations generated by AI models complied with the guideline recommendations was investigated item by item. To evaluate the robustness of the guideline-compliance coding, a secondary rater (T.O.K.) independently coded a subset of the menus. A total of 54 menus (six patients per three postoperative phase × three AI models) were randomly selected and coded by the second evaluator using the same binary checklist.

### 2.4. Statistical Analysis

To calculate average daily energy and macro- and micronutrient contents, all menu plans generated by ChatGPT-4o, Grok-3, and DeepSeek were entered into the Nutrition Information System (Ebispro for Windows, Stuttgart, Germany; Turkish version/BeBiS 8.1). Statistical analyses were conducted using the Statistical Package for the Social Sciences (version 30.0; IBM Corp., Armonk, NY, USA). The primary outcome of the study was the Total Guideline Compliance Score, a composite metric reflecting the extent to which each AI model adhered to essential clinical recommendations for early post-SG nutrition. All nutrient-based comparisons—including energy, macronutrient, and micronutrient values—were initially considered exploratory. Normality was assessed using the Kolmogorov–Smirnov test, and all continuous variables exhibited non-normal distributions; therefore, nonparametric methods were applied. Overall differences among the three AI models were evaluated using the Friedman test, and significant findings were followed by Wilcoxon signed-rank pairwise comparisons. To control for Type I error inflation, Bonferroni correction was applied, yielding an adjusted significance threshold of *p* < 0.016 (0.05/3). Descriptive statistics included the mean, 95% confidence intervals, median, and 25th–75th percentiles. Additionally, the observed proportions for each guideline item were calculated, and 95% confidence intervals for these proportions were estimated using the Wilson score method for binomial distributions. Inter-rater reliability for the total guideline-compliance scores was examined using Intraclass Correlation Coefficients (ICC).

Because LLMs often generate highly similar or partially templated outputs in response to standardized prompts, the 40 plans within each model–phase cannot be treated as fully independent observations. This introduces pseudo-replication, reduces the effective sample size, and limits the interpretability of inferential statistics. Moreover, performing simultaneous comparisons across a wide range of nutrient variables increases the likelihood of Type I error due to the multiplicity of exploratory tests. For these reasons, formal hypothesis testing was intentionally restricted to three clinically critical nutritional variables—energy, protein, and thiamine—together with the study’s primary outcome, the guideline compliance scores. All remaining macro- and micronutrient outcomes were reported descriptively to minimize excessive multiple testing. This analytical strategy was designed to characterize general model-level behavior within each postoperative phase rather than performing patient-level hypothesis testing. Consequently, all *p*-values in this study should be interpreted with caution given the dependence structure inherent to AI-generated outputs and the resulting reduction in effective sample size.

## 3. Results

### 3.1. Evaluation of Menu Plans in Terms of Guideline Recommendations

**Figure 1:**
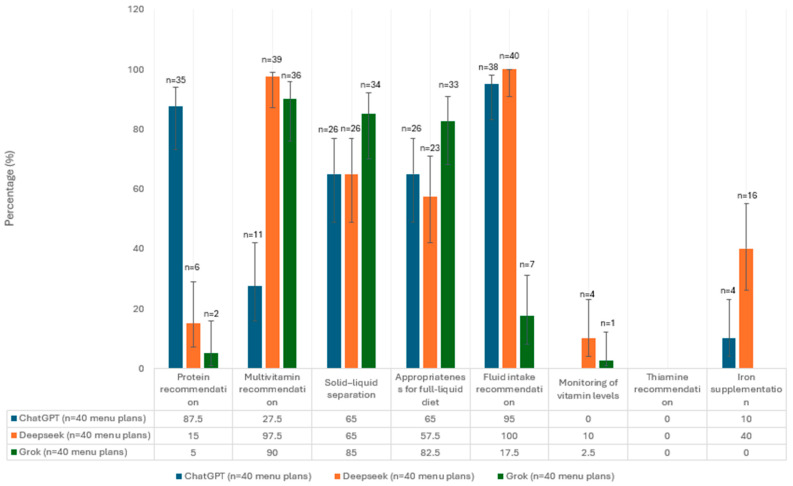
The percentage and confidence intervals of liquid-phase menus in which each guideline recommendation was present.

Nutrition recommendations for the early post-surgery period provided by the AI platforms (ChatGPT, DeepSeek AI, and Grok) were evaluated according to a checklist developed on the basis of current metabolic and bariatric surgery guidelines (ASMBS/AACE/TOS). The percentage of nutrition recommendations for the liquid phase that each AI platform met is depicted in Figure 1.

ChatGPT demonstrated the highest compliance rates in key criteria, including fluid intake (95.0%), and protein intake recommendations (87.5%), specifically for the liquid phase. However, the recommendation rate was low for critical micronutrients and complication management topics, including multivitamin supplementation (27.5%), thiamine intake recommendation (0%), iron supplementation (10%). Multivitamin supplementation (97.5%), adequate fluid intake (100%) and iron supplementation (40%) were the topics most frequently recommended by DeepSeek AI. Protein intake recommendations were only provided at (15%). Recommendations for solid–liquid separation (65%) were moderate, whereas the recommendation rate for suitability for a full liquid diet was 57.5%. In the liquid phase nutrition plans generated by Grok, the most frequently recommended components suitability for a full liquid diet (82.5%), and solid–liquid separation (85%). However, protein recommendations were only provided in 5% of cases, whereas multivitamin supplementation recommendations were at 90%. Several elements including monitoring vitamin levels (2.5%), and thiamine intake recommendations (0%), were noted at very low rates and iron supplementation was not recommended at all.

**Figure 2:**
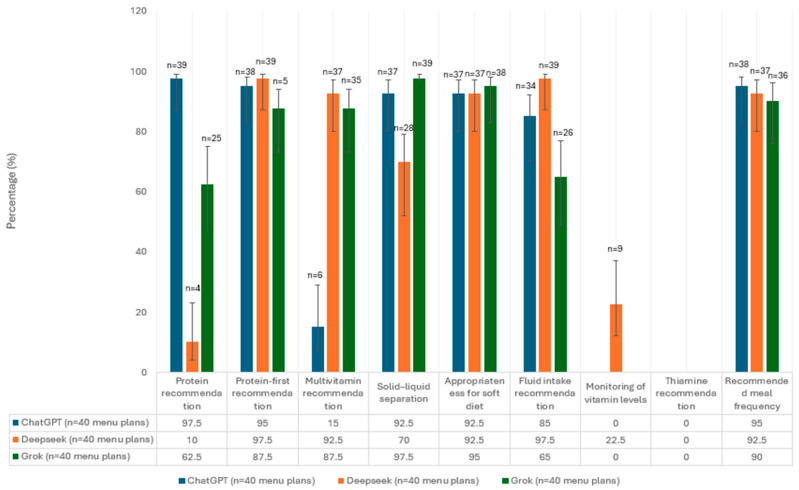
The percentage and confidence intervals of pureed-phase menus in which each guideline recommendation was present.

Investigating the frequency of recommendations for the puree stage revealed differences across AI platforms. Although ChatGPT provided the highest rate (97.5%) of protein intake recommendations, DeepSeek AI did not include protein intake recommendations in most of the programs it generated for this stage. The priority of protein consumption was highlighted at a high rate by all platforms (87.5–97.5%); however, the multivitamin supplement recommendation was only present at a low rate in ChatGPT (15%). Solid–liquid separation and puree-period-specific high-protein food recommendations were frequently reported on all three platforms. DeepSeek AI most frequently provided fluid intake recommendations (97.5%). The percentage of nutrition recommendations for the puree period provided by each AI platform is shown in Figure 2.

**Figure 3:**
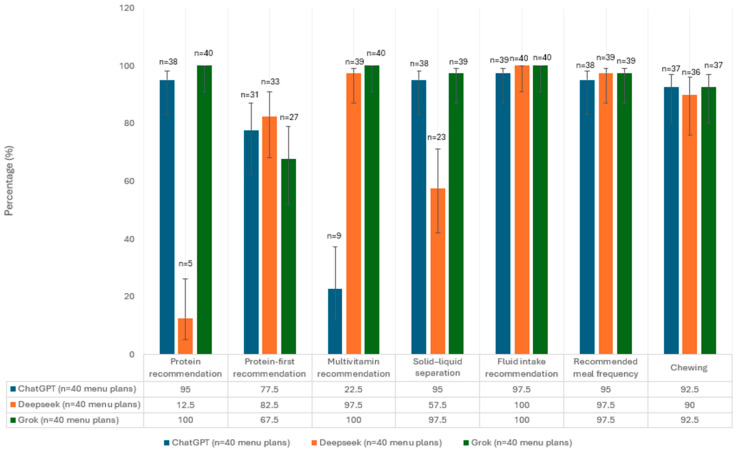
The percentage and confidence intervals of solid-phase menus in which each guideline recommendation was present.

Analyzing the percentages of recommendations for the solid phase (day 35) indicated that Grok and ChatGPT provided protein intake recommendation at high rates (100% and 95%, respectively), whereas DeepSeek AI generated this recommendation at a relatively low rate (12.5%). The multivitamin supplement recommendation was indicated at almost full rates (100% and 97.5%) in the programs generated by Grok and DeepSeek AI for almost all patients, whereas ChatGPT recommended it at only 22.5%. Grok and ChatGPT indicated the solid–liquid distinction at a high rate (97.5% and 95%, respectively), whereas DeepSeek AI provided this recommendation at 57.5%. The fluid intake recommendation was relatively high across all platforms (97.5–100%). Protein consumption priority was recommended at a medium-high level across all platforms (DeepSeek AI, 82.5%; ChatGPT, 77.5%; and Grok, 67.5%). The meal recommendation was stated relatively extensively across all platforms (95–97.5%). Similarly, all three platforms recommended increasing the number of chews at rates > 90%. The percentage of nutrition recommendations related to the strict phase that each AI platform provided is depicted in Figure 3.

**Figure 4:**
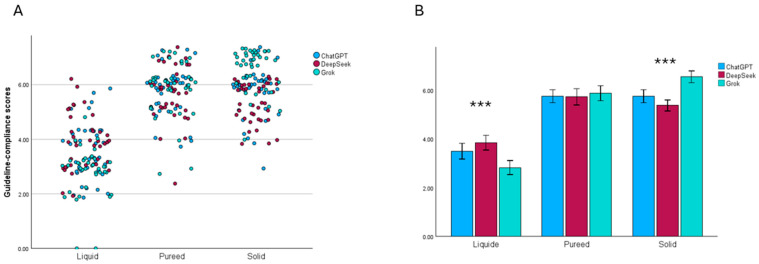
Comparison of guideline-compliance scores generated by ChatGPT, DeepSeek, and Grok across the liquid, puréed, and solid diet phases. (**A**) Distribution of individual guideline-compliance scores across all participants for each model and diet phase. Each point represents a single score, illustrating the variability within and across models. (**B**) Mean guideline-compliance scores with 95% confidence intervals for each model across the three diet phases. Significant model differences within diet phases are indicated (*** *p* < 0.001). Bonferroni-adjusted pairwise comparisons showed that significant differences occurred between Grok and DeepSeek.

The compliance scores of the menu plans generated by the three AI models (ChatGPT, DeepSeek, and Grok) were examined in Figure 4. For all models, overall compliance scores were lowest in the liquid phase, while higher and comparable scores were observed in the puree and solid phases. Bonferroni-corrected pairwise comparison tests revealed significant differences between the models in the liquid and solid phases (*p* < 0.001). In the liquid phase, the Grok model had significantly lower compliance scores than DeepSeek (*p* < 0.001). In contrast, in the solid phase, Grok achieved the highest compliance score, outperforming DeepSeek with a statistically significant margin (*p* < 0.001). No significant differences were identified among the three models in the puree phase (*p* = 0.179).

To assess the reproducibility of the scoring procedure, two raters independently coded a subset of 54 diet menus. Inter-rater reliability for the total guideline-compliance scores was evaluated using a two-way mixed-effects ICC with absolute agreement. Inter-rater reliability for the total guideline-compliance scores was good, with an ICC of 0.776 (95% CI: 0.637–0.866), indicating strong absolute agreement between raters (Supplementary File).

### 3.2. Evaluation of the Energy and Macronutrient Contents of Menu Plans Based on AI Platforms

In our study, 40 patients were simulated, encompassing 20 females and 20 males with an average age of 36.7 ± 11.9 years and an average BMI of 40.1 ± 3.6 kg/m^2^. For these 40 simulated patients, 360 diet plans scheduled by ChatGPT, DeepSeek AI, and Grok for days 5, 16, and 35 post-SG were analyzed. Analyzing the energy content of the diet programs revealed that the energy levels of programs planned by Grok were lower than those generated by the other platforms in all three periods (*p* < 0.001). A similar situation was noted for protein (g) (*p* < 0.001). Analyzing macronutrient percentages indicated that the median protein percentage was the highest on ChatGPT at 34.0% (29.2–42.0%) on day 5, whereas it was the highest on Grok at on days 16 [38.0% (31.0–40.0%)] and 35 days [40.0 (35.2–46.0)]. Fat percentages did not differ across platforms on day 5. On day 35, ChatGPT [47.0 (43.0–49.0)] and DeepSeek AI [47.0 (39.0–58.0)] exhibited similarities, whereas the fat per-centage of Grok [36.5 (30.0–43.0)] was lower. Analyzing the carbohydrate percentage, a similarity was observed across all three platforms on day 35. On day 5, the lowest percentage was 38.5% (30.0–50.5%) for ChatGPT, whereas on day 16, the lowest per-centage was 21.0% (14.0–27.7%) for DeepSeek AI. The highest fiber intake for all three periods was recorded by DeepSeek AI. Cholesterol levels were comparable for all three periods by DeepSeek AI and ChatGPT. A comparison of the energy and macronutrient elements of the menu plans according to the AI platforms is presented in Table 3.

### 3.3. Evaluation of the Micronutrient Content of Menu Plans Based on AI Platforms

The micronutrient content of diet plans generated by AI platforms (ChatGPT, DeepSeek AI, and Grok) is compared in Table 4. For vitamin B1 content, ChatGPT and DeepSeek AI provided similar levels on day 5, whereas the menus generated by Grok contained significantly lower vitamin B1 amounts (*p* < 0.001). According to the analyses, DeepSeek AI generated the highest iron and zinc values across all three periods, while folate intake was highest on days 16 and 35 for DeepSeek AI and on day 5 for ChatGPT. Analysis of vitamin C content showed DeepSeek AI provided the highest vitamin C levels on day 5, whereas on day 35, ChatGPT generated the highest values. Calcium levels were highest in the menus generated by ChatGPT on days 5 and 35, whereas DeepSeek AI produced the highest calcium content on day 16.

### 3.4. Sensitivity Analysis of Repeated Menu Generation

**Figure 5:**
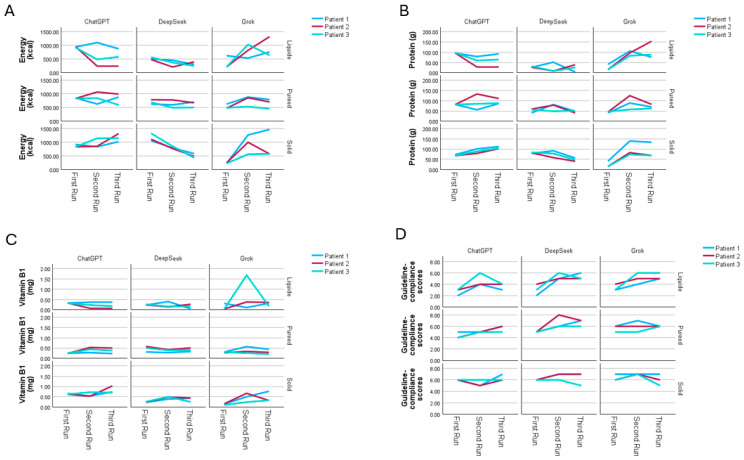
Sensitivity analysis of model-generated menus across three repeated runs using three representative patients. Panels (**A**–**D**) illustrate the variability observed in (**A**) energy (kcal), (**B**) protein (g), (**C**) thiamine (mg), and (**D**) guideline-compliance scores across repeated menu-generation attempts. This sensitivity analysis was conducted using three representative patients across three models (ChatGPT, DeepSeek, Grok) and three phases (liquid, puree, solid). Lines represent the First, Second, and Third Run for each patient within each model–phase combination.

A supplemental sensitivity analysis was conducted using a smaller subset of three representative patients across three dietary phases and three models to evaluate the extent of run-to-run variability in model-generated menus (Figure 5). Energy, protein, thiamine, and guideline-compliance scores exhibited noticeable fluctuations across the first, second, and third runs within the same patient–model-phase combination. The magnitude of variation differed by nutrient and by model. For example, in the liquid phase under the Grok model, Patient 2 showed considerable run-to-run variation in energy values, with measurements of 214.32, 821.52, and 1320.87 kcal across the three runs. Regarding protein, Patient 2 in the puree phase demonstrated variability under the DeepSeek model, where protein content increased from 45.04 g to 124.58 g in the second run, followed by a decrease to 82.22 g in the third run. Thiamine content remained relatively stable across most model–phase combinations; however, a marked deviation was observed for Patient 3 in the liquid phase under the Grok model, where thiamine increased from 0.04 mg to 1.68 mg in the second run. Guideline-compliance scores also showed modest run-to-run differences.

## 4. Discussion

Sleeve gastrectomy is a widely used metabolic and bariatric procedure, and its success is closely linked to adherence to postoperative nutritional protocols [4]. The potential use of AI technology in nutritional recommendations has recently attracted significant interest [9,15,16]. In this context, this study compared the energy, macro-, and micronutrient contents of menus generated by AI-based diet planning platforms (ChatGPT-4o, Grok-3, and DeepSeek) for the early period following SG with their level of compliance with current metabolic and bariatric surgery guidelines. The total diet programs planned for 40 simulated individuals were analyzed; the results revealed that, under the specific prompts used in this study and the model versions available at the time of testing, Grok systematically generated diet plans with lower energy and macronutrient values, whereas DeepSeek AI provided plans with the highest content for several micronutrients. ChatGPT frequently generated higher protein ratios and offered the highest compliance with guidelines for the liquid and puree phases. However, all platforms demonstrated deficiencies regarding critical micronutrient recommendations (thiamine, iron, and multivitamins) and information on complications. These findings should be interpreted within the context of the specific model versions and testing conditions used in this study, as updates to large language models can rapidly alter their behavior and may lead to different outputs in future iterations. These findings reveal the potential and significant limitations of AI platforms in postoperative nutrition management. Our study investigated feeding programs generated by three different LLMs for various simulated profiles of patients post-SG. This study also functions as a controlled stress test designed with minimal inputs, evaluating the behavior of LLMs in the context of postoperative nutrition planning after metabolic and bariatric surgery under hypothetical and structured conditions.

Clinical guidelines have recommended starting a clear liquid diet within the first 24 h postoperatively, followed by a 2–4-week process of transitioning to full liquids, pureed foods, and subsequently soft/chewable foods [17,18,19]. Gradually progressing food consistency is crucial to supporting gastrointestinal tolerance and ensuring nutritional safety [20]. To successfully manage this process, patients are recommended to receive tailored counseling from a metabolic and bariatric dietitian experienced in postoperative nutrition before discharge [21,22]. However, AI-based nutrition programs may not always clearly demonstrate how the postoperative dietary progression through the liquid, puréed, and solid phases should be structured. In particular, determining the appropriate timing for transitioning from liquids to puréed foods and from puréed to solid foods requires a systematic evaluation of several factors, including the patient’s clinical condition, individual tolerance, and nutrition education. Therefore, the development of postoperative dietary recommendations should be carried out under the supervision of a specialist dietitian.

The analysis of menu outputs generated by the AI models revealed substantial variation in energy and macronutrient distributions, along with notable inconsistencies across platforms. Grok produced the lowest energy and protein contents in all postoperative stages, whereas DeepSeek AI provided higher energy and protein values, particularly on Days 16 and 35. ChatGPT-4o demonstrated a more balanced and relatively stable profile among the three models. Similarly, two independent simulation studies conducted in the contexts of diabetes and MASLD have reported that LLMs show inconsistencies in achieving energy and macronutrient targets [9,16].

Rapid weight loss is observed in the first months following metabolic and bariatric surgery, and this is frequently associated with unwanted decreases in lean body mass and muscle mass [23]. Daily protein intake of >60 g has been associated with lower lean mass loss and therefore has a protective effect [24]. Current guidelines have recommended a minimum of 60 g/day of protein in the post-metabolic and bariatric surgery period, up to 1.5 g/kg/day based on the ideal body weight, noting that this amount can be increased to 2.1 g/kg/day in some individuals [22,25]. Studies have demonstrated that whey protein supplementation significantly increases protein intake and, when combined with resistance exercise, may limit lean body mass loss by supporting muscle strength [26,27]. In our study, when the macronutrient content of the AI platform-generated menu plans was analyzed, ChatGPT remained above the recommended minimum protein level (≥60 g/day) across all periods, whereas DeepSeek AI met this target except during the liquid phase (day 5). In contrast, the Grok-generated menu plans had low protein content across all periods and remained below the target. These findings indicate that AI-based plans show meaningful inter-platform differences in protein adequacy, and that such variations may imply potential risks for postoperative muscle mass loss based on nutrient shortfalls; however, these risks are not confirmed by patient outcomes or body composition data. Clinicians will need to provide specific patient-driven prompts regarding protein goals to ensure that any AI model generates menus with adequate protein content.

To prevent long-term complications, postoperative nutritional monitoring in patients who have undergone metabolic and bariatric surgery is crucial [28]. Serum vitamin levels are often low in patients with obesity even before surgery [29]. Procedures, including gastric bypass, SG, and biliopancreatic diversion, alter the anatomical and functional structure of the gastrointestinal system, negatively influencing the absorption of several micronutrients, including iron, calcium, B1, B9 (folate), B12, and vitamin D [30]. This situation can lead to serious health issues including anemia, osteopenia, and neurological symptoms [30,31]. Therefore, nutritional recommendations provided in the early stages should encompass not only macronutrients but also micronutrient supplementation and vitamin level monitoring [28]. However, the findings of our study revealed that the three major LLM-based AI platforms demonstrated inconsistent performance in terms of micronutrient monitoring and supplementation. According to liquid-phase data, ChatGPT rarely provided critical recommendations, including multivitamin, thiamine, and iron supplementation. DeepSeek AI demonstrated strong performance in recommending multivitamin supplementation. The puree and solid phases showed a similar trend; ChatGPT generally excelled in fundamental areas, such as protein intake and patient education, but fell short in clinically critical areas, such as micronutrient supplementation and tailored monitoring. DeepSeek AI yielded high rates, particularly in multivitamin recommendations and meal planning. Grok generally generated comprehensive content in solid phase recommendations; however, it fell short in protein and micronutrient recommendations during the puree and liquid phases. Clinicians should provide individualized, patient-centered prompts addressing protein, micronutrients, and other key dietary considerations when generating menus for patients using any AI model.

In general, patients may experience volume depletion and dehydration as they often struggle to consume adequate amounts of fluid while adapting to their reduced gastric capacity [32,33]. Although all three models included statements related to fluid intake, ChatGPT-4o incorporated such recommendations in the vast majority of menus across all phases. DeepSeek AI and Grok-3, by contrast, provided fluid-intake reminders at lower frequencies during the liquid and purée phases, while both models included this recommendation in nearly all menus during the solid phase; moreover, this finding aligns with a previous observation regarding the ability of AI models to interpret context and guide users toward appropriate information [34]. These findings are limited to the specific model versions and testing conditions used in this study, and updates to large language models may lead to different outcomes in future iterations.

Postoperatively, to maximize the effectiveness of metabolic and bariatric surgery and minimize the risk of weight regain, patients should receive regular counseling from a registered dietitian [35]. Detailed preoperative nutrition education, appropriate patient selection, and postoperative nutritional follow-up with patient compliance to guidelines can prevent the risk of metabolic and bariatric surgery-associated malnutrition [36]. Analyzing the LLM-generated nutrition programs in our study revealed that all three AI platforms underscored dietitian follow-up and approval in their recommendations across all periods. Although LLMs have been shown to provide high accuracy for certain nutrition-related questions [37], bariatric surgery is a high-risk field that requires strict adherence to clinical protocols, and issues such as critical nutrient recommendations, complication management, and the timing of dietary phase transitions necessitate the specialized knowledge and expertise of dietitians and other healthcare professionals, particularly given the potential postoperative clinical challenges [32].

This study offers several noteworthy strengths. First, it represents one of the earliest simulation-based investigations comparing the performance of three large language models (ChatGPT-4o, Grok-3, and DeepSeek AI) in generating early postoperative nutrition plans following sleeve gastrectomy. The study adopts a comprehensive and multidimensional evaluation framework, assessing not only the energy, macro- and micronutrient composition of AI-generated menus but also their alignment with international metabolic and bariatric surgery guidelines (AACE/TOS/ASMBS). Moreover, evaluating each model across the three distinct phases of the early postoperative period (liquid, puréed, and solid) provides insight into how LLMs respond to phase-specific nutritional requirements.

This study’s sensitivity analysis revealed that even when the same prompt was repeated three times, all large language models (LLMs) demonstrated notable run-to-run variability in outputs related to energy, protein, micronutrients, and guideline compliance. Similarly, another study reported significant temporal fluctuations in energy and macronutrient percentages when identical prompts were re-entered into ChatGPT-4o at different time points [15]. The effects of temporal inconsistencies in LLM outputs on user experience and model reliability are still not fully understood [38]. The fact that models produced inconsistent outputs across diet phases despite receiving identical inputs—particularly evident in Grok’s energy and micronutrient estimations in the liquid phase and DeepSeek’s late-phase protein predictions—highlights the inherently stochastic nature of LLMs and their limited reproducibility. Therefore, caution is warranted when using AI-generated postoperative nutrition plans in clinical practice, and these outputs should always be evaluated by an expert bariatric dietitian for consistency and safety.

However, this study has several limitations. First, given that LLMs are continuously updated and their behavior is not entirely fixed, ChatGPT-4o, DeepSeek AI, and Grok-3 may produce different responses to identical prompts in the future [39]. This highlights that our findings are bounded by the specific model versions used and the time period during which they were tested. Second, as this study is based on a simulation-driven design, the findings do not incorporate real patient data, behavioral responses, or clinical outcomes, which may limit external validity. Therefore, the results should be interpreted within the inherent constraints of a simulation framework. Additionally, the prompts used in the study were standardized and did not include individualized clinical information such as patients’ medications, comorbidities, metabolic conditions, or other medical variables. Consequently, the evaluation reflects optimal and controlled conditions based on hypothetical profiles and may not fully capture the clinical heterogeneity observed in real patients. Furthermore, because all prompts were delivered in Turkish, the results may not reflect the multilingual performance of the models or potential biases that might emerge in other languages. Finally, although users typically interact with LLMs through a sequence of iterative prompts rather than a single query, this study evaluated only the models’ initial outputs. Yet, unlike traditional web searches, LLMs can engage in multi-turn dialogs, allowing users to request clarification and refine responses step by step.

Our findings suggest that LLMs may serve as a potential supplementary resource in postoperative metabolic bariatric nutrition when used under appropriate professional supervision. Given the increasing reliance on online sources for nutrition information [39,40], evaluating the potential role and limitations of LLM-based systems in this field has become increasingly important. However, the scope of our study is limited, as the models were assessed through a single, standardized stress test, and the results may not fully reflect their performance across diverse clinical scenarios. Future research could examine the consistency of LLM outputs with metabolic and bariatric nutrition guidelines across broader conditions and explore how clinical variability—such as comorbidities, medication use, or cultural dietary practices—affects their ability to generate individualized recommendations. Investigating the impact of linguistic and cultural differences on model performance would also be valuable. Finally, defining how these tools can be used within postoperative metabolic and bariatric nutrition and the extent of professional oversight required will be essential for their safe and appropriate integration into clinical workflows.

## 5. Conclusions

This study provides a comparative evaluation of guideline alignment among three large language models—ChatGPT-4o, Grok-3, and DeepSeek AI—in generating early postoperative nutrition recommendations following sleeve gastrectomy. The findings indicate that the models offer recommendations at varying levels across the liquid, puréed, and solid phases, with notable differences in phase-specific requirements. In the liquid phase, ChatGPT-4o demonstrated higher alignment with core recommendations, whereas DeepSeek AI and Grok-3 exhibited comparatively lower levels of recommendation provision. The models showed more similar patterns during the puréed phase, while Grok-3 demonstrated higher recommendation rates in the solid phase. Statistical analyses confirmed significant differences between the models, particularly in the liquid and solid phases.

The findings of this study reflect the behavior of large language models under controlled and narrowly scoped stress-test conditions and do not provide direct evidence of clinical effectiveness. The evaluated meal plans were based on single-round outputs generated by ChatGPT-4o, DeepSeek AI, and Grok-3 for standardized patient profiles; therefore, these outputs are not suitable for clinical application without real-world data, patient feedback, and oversight by qualified professionals. Future research incorporating patient-level data, clinical outcome measures, and multilingual validation will be essential to determine the true clinical utility of AI-assisted dietary planning tools in postoperative bariatric nutrition care. In addition, for AI models to generate accurate and safe nutrition plans, clinicians must provide specific, individualized patient-driven prompts regarding protein, micronutrients, and other key areas of concern.

## Figures and Tables

**Table 1:** Guideline Recommendations from the 2019 AACE/TOS/ASMBS Clinical Practice Guidelines.

Guidance Data
A minimal protein intake of 60 g/d and up to 1.5 g/kg ideal weight per day should be adequate; higher amounts of protein intake—up to 2.1 g/kg ideal weight per day—need to be assessed on an individualized basis (Grade D).
A low-sugar, clear-liquid meal program can usually be initiated within 24 h after any of the surgical bariatric procedures, but this diet and meal progression should be discussed with the surgeon and guided by the registered dietician (RD) (Grade C; BEL 3).
A consultation for postoperative meal initiation and progression must be arranged with an RD who is knowledgeable about the postoperative bariatric diet (Grade A; BEL 1).
Patients should receive education in a protocol-derived staged meal progression based on their surgical procedure (Grade D).
Patients should be counseled to eat 3 small meals during the day and chew small bites of food thoroughly before swallowing (Grade D).
Patients should be counseled about the principles of healthy eating, including at least 5 daily servings of fresh fruits and vegetables (Grade D).
Protein intake should be individualized, assessed, and guided by an RD regarding sex, age, and weight (Grade D).
Minimal daily nutritional supplementation for patients with BPD with or without DS, RYGB, and SG should be in chewable form initially and then as 2 adult multivitamins plus minerals (each containing iron, folic acid, and thiamine) (Grade B; BEL 2).
2000–3000 IU/day of vitamin D should be administered, titrated to maintain 25(OH)D levels > 30 ng/mL. (Grade A; BEL 1).
Total iron as 18 to 60 mg via multivitamins and additional supplements (Grade A, BEL 1).
Vitamin B12 (parenterally as sublingual, subcutaneous, or intramuscular preparations; orally if determined to be adequately absorbed) (Grade B; BEL 2).
Once patients can tolerate orals, fluids should be consumed slowly, preferably at least 30 min after meals to prevent GI symptoms, and in sufficient amounts to maintain adequate hydration (>1.5 L daily) (Grade D).
The postoperative follow-up checklist includes maintaining adequate hydration (usually >1.5 L/day orally); 24 h urinary calcium excretion at 6 months and then annually; annual vitamin B_12_ monitoring (with optional MMA and homocysteine testing, and every 3–6 months if the patient is receiving supplementation); assessment of folic acid (with optional RBC folate), iron studies, 25-hydroxyvitamin D, and iPTH; vitamin A measurement initially and then every 6–12 months; evaluation of copper, zinc, and selenium when clinically indicated; and thiamine assessment in the presence of relevant symptoms or risk factors.
All post-WLS patients should take at least 12 mg thiamine daily (Grade C; BEL 3).

These guidelines are endorsed by the American Society for Nutrition (ASN), the Obesity Action Coalition (OAC), the International Federation for the Surgery of Obesity and Metabolic Disorders (IFSO), the International Society for the Perioperative Care of the Obese Patient (ISPCOP), and the American Society for Parenteral and Enteral Nutrition (ASPEN). SG = sleeve gastrectomy; RYGB = Roux-en-Y gastric bypass; BPD/DS = biliopancreatic diversion with duodenal switch; RD = registered dietitian; MMA = methylmalonic acid; RBC = red blood cell; iPTH = intact parathyroid hormone.

**Table 2:** Definitions and Coding Rules for the Metabolic and Bariatric Nutrition Guideline Compliance Checklist.

Suggestion	Period	Explanation	Present (1 Score)	Absent (0 Score)
Appropriateness for full-liquid diet	Liquid	If the menu provided for Day 5 consists solely of liquids.		
Iron supplementation	Liquid	Use a multivitamin with iron.		
Appropriateness for soft diet	Pureed	List of soft, easy-to-chew foods		
Chewing	Solid	Bites should be thoroughly chewed		
Protein-first recommendation	Pureed, solid	During the solid phase, start each meal with protein to meet your needs.		
Recommended meal frequency	Pureed, solid	Consume 3 small meals.		
Monitoring of vitamin levels	Liquid, pureed	Have your vitamin and mineral levels checked regularly.		
Thiamine recommendation	Liquid, pureed	Use multivitamin with thiamine		
Protein recommendation	Liquid, pureed, solid	Aim for at least 60 g of protein per day		
Multivitamin recommendation	Liquid, pureed, solid	Take a daily multivitamin. A single vitamin recommendation does not constitute a multivitamin.		
Solid–liquid separation	Liquid, pureed, solid	Stop fluids 30 min before and after meals		
Fluid intake recommendation	Liquid, pureed, solid	Aim for at least 1.5 L fluids daily.		
		Maximum Score		
		Liquid	8	
		Pureed	9	
		Solid	7	

**Table 3:** Comparison of macronutrients in menu plans according to artificial intelligence platforms.

		ChatGPT	DeepSeek	Grok	*p*
**Energy** **(kcal)**	**Day 5**	831.4 (655.3–926.3)	742.1 (530.9–877.7)	240.4 (214.3–246.9)	**<0.001 ^β,†^**
	**Day 16**	833.1 (731.7–893.4)	1015.8 (810.1–1073.5)	532.4 (467.7–636.4)	**<0.001 ^α,β,†^**
	**Day 35**	978.8 (825.1–1131.3)	1063.3 (972.2–1235.3)	730.4 (306.6–805.9)	**<0.001 ^α,β,†^**
**Protein (g)**	**Day 5**	70.2 (53.3–73.6)	41.6 (27.4–49.5)	19.6 (15.6–19.7)	**<0.001 ^α,β,†^**
	**Day 16**	71.1 (59.7–81.4)	74.9 (64.2–85.7)	47.1 (45.2–49.5)	**<0.001 ^β,†^**
	**Day 35**	71.6 (66.8–82.8)	77.4 (72.6–83.5)	69.6 (35.8–70.1)	**<0.001 ^β,†^**
**Protein (%)**	**Day 5**	34.0 (29.2–42.0)	23.0 (14.3–27.5)	33.0 (28.0–33.0)	
	**Day 16**	37.0 (30.2–40.0)	32.0 (26.2–36.0)	38.0 (31.0–40.0)	
	**Day 35**	31.0 (29.0–34.0)	28.0 (27.0–31.0)	40.0 (35.2–46.0)	
**Fat (g)**	**Day 5**	19.6 (18.1–25.0)	18.6 (12.3–25.7)	6.5 (3.4–6.6)	
	**Day 16**	36.3 (26.2–42.9)	43.9 (29.8–69.5)	17.2 (14.9–19.0)	
	**Day 35**	48.8 (43.3–59.2)	59.7 (42.8–68.2)	31.5 (10.2–37.6)	
**Fat (%)**	**Day 5**	22.5 (19.0–28.2)	22.5 (14.3–33.0)	24.0 (19.0–24.75)	
	**Day 16**	40.0 (34.2–43.7)	43.5 (36.5–59.0)	31.0 (25.0–34.7)	
	**Day 35**	47.0 (43.0–49.0)	47.0 (39.0–58.0)	36.5 (30.0–43.0)	
**CHO (g)**	**Day 5**	78.1 (41.5–96.9)	94.4 (56.4–116.8)	25.7 (22.5–28.7)	
	**Day 16**	46.3 (34.8–61.4)	49.3 (35.5–54.1)	40.0 (32.6–68.1)	
	**Day 35**	52.1 (36.3–62.1)	59.4 (36.9–77.4)	30.4 (20.4–56.2)	
**CHO (%)**	**Day 5**	38.5 (30.0–50.5)	46.5 (38.3–69.0)	43.0 (41.0–59.8)	
	**Day 16**	24.0 (18.2–30.2)	21.0 (14.0–27.7)	31.5 (26.2–44.7)	
	**Day 35**	21.0 (17.0–26.0)	22.0 (14.5–29.0)	25.5 (16.0–29.7)	
**Fiber (g)**	**Day 5**	2.4 (1.3–4.8)	3.3 (2.8–7.2)	0.47 (0.45–0.56)	
	**Day 16**	5.6 (3.2–6.8)	12.6 (8.5–13.3)	4.4 (3.2–4.9)	
	**Day 35**	8.2 (6.6–9.7)	14.4 (13.0–17.6)	4.1 (2.3–4.4)	
**Cholesterol (mg)**	**Day 5**	47.8 (40.0–59.6)	65.0 (28.7–91.49	12.0 (6.0–13.0)	
	**Day 16**	363.8 (126.2–451.0)	235.6 (204.9–442.5)	96.1 (92.1–136.0)	
	**Day 35**	432.1 (401.0–464.5)	408.9 (379.2–426.1)	429.4 (356.7–439.3)	

Data were presented as median (25th–75th percentile). Comparisons among the three models were performed using the Friedman test, and pairwise comparisons for significant results were evaluated using the Wilcoxon signed-rank test. Statistically significant values were highlighted in bold. Multiple comparisons were controlled using the Bonferroni correction, and a *p*-value < 0.016 was considered statistically significant. ^α^: Significant difference between ChatGPT and DeepSeek; ^β^: Significant difference between ChatGPT and Grok; ^†^: Significant difference between DeepSeek and Grok.

**Table 4:** Comparison of micronutrients in menu plans according to artificial intelligence platforms.

		ChatGPT	DeepSeek	Grok	*p*
**Vitamin B1 (mg)**	**Day 5**	0.33 (0.29–0.36)	0.33 (0.21–0.44)	0.11 (0.08–0.10)	**<0.001 ^β,†^**
	**Day 16**	0.38 (0.32–0.46)	0.57 (0.49–0.62)	0.29 (0.28–0.31)	**<0.001 ^α,β,†^**
	**Day 35**	0.62 (0.52–0.65)	0.67 (0.58–0.71)	0.35 (0.17–0.36)	**<0.001 ^α,β,†^**
**Folic acid (µg)**	**Day 5**	79.3 (61.7–86.8)	74.2 (53.5–103.9)	21.6 (16.5–23.2)	
	**Day 16**	104.1 (93.8–132.6)	145.1 (131.4–168.8)	82.4 (61.2–94.4)	
	**Day 35**	172.8 (148.7–188.6)	176.4 (148.2–209.1)	110.5 (89.1–131.9)	
**Vitamin C** **(mg)**	**Day 5**	28.1 (18.8–49.0)	42.7 (26.7–63.8)	12.8 (10.9–12.9)	
	**Day 16**	38.3 (29.4–47.4)	47.9 (35.1–57.5)	47.0 (23.2–54.4)	
	**Day 35**	63.9 (57.8–70.8)	45.8 (38.1–60.2)	38.7 (22.3–42.3)	
**Calcium** **(mg)**	**Day 5**	676.0 (598.1–861.1)	519.6 (394.7–745.9)	244.0 (158.1–244.0)	
	**Day 16**	641.7 (567.6–875.1)	769.6 (665.2–828.7)	397.1 (324.8–415.6)	
	**Day 35**	849.8 (689.1–943.1)	824.3 (684.7–1060.4)	416.3 (376.6–476.3)	
**Iron** **(mg)**	**Day 5**	2.1 (1.4–2.6)	2.4 (1.5–3.4)	0.52 (0.44–0.55)	
	**Day 16**	3.0 (2.4–3.3)	4.7 (3.5–5.6)	1.6 (1.5–2.7)	
	**Day 35**	4.2 (3.6–5.1)	5.8 (4.9–6.2)	2.8 (1.5–2.9)	
**Zinc** **(mg)**	**Day 5**	3.4 (2.7–4.0)	2.9 (2.0–6.8)	0.95 (0.85–1.3)	
	**Day 16**	4.5 (3.2–4.8)	4.9 (4.3–5.7)	1.9 (1.6–2.5)	
	**Day 35**	5.9 (4.9–7.6)	6.2 (5.3–7.4)	3.7 (1.6–3.8)	

Data were presented as median (25th–75th percentile). Comparisons among the three models were performed using the Friedman test, and pairwise comparisons for significant results were evaluated using the Wilcoxon signed-rank test. Statistically significant values were highlighted in bold. Multiple comparisons were controlled using the Bonferroni correction, and a *p*-value < 0.016 was considered statistically significant. ^α^: Significant difference between ChatGPT and DeepSeek; ^β^: Significant difference between ChatGPT and Grok; ^†^: Significant difference between DeepSeek and Grok.

## Data Availability

The study materials have been deposited in an open-access repository (Open Science Framework; OSF) and are publicly available at https://osf.io/pkdzq/overview, accessed on 30 November 2025.

## Supplementary Materials

The following supporting information can be downloaded at: https://www.mdpi.com/article/10.3390/nu17243957/s1, Table S1: Raw rater scores for 54 menus (Rater 1 and Rater 2); Table S2: Inter-rater reliability for total guideline-compliance scores across all menus (N = 54).
