# Supplementary material for: Guideline Compliance of Artificial Intelligence–Generated Diet Plans After Bariatric Surgery: A Cross-Sectional Simulation Comparing ChatGPT-4o, DeepSeek and Grok-3
Source: Nutrients. 2025 Dec 18;17(24):3957. doi: 10.3390/nu17243957 (PMC12735798; doi:10.3390/nu17243957)
Supplement: Supplementary file 1 [file nutrients-17-03957-s001.zip › nutrients-4004350-supplementary.pdf]

Supplementary Table S1. Raw rater scores for 54 menus (Rater 1 and Rater 2).

| Phase | Model | Rater 1 Score | Rater 2 Score |
|---|---|---|---|
| Liquid | ChatGPT | 2.00 | 2.00 |
| Liquid | DeepSeek | 2.00 | 4.00 |
| Liquid | Grok | 3.00 | 3.00 |
| Liquid | ChatGPT | 2.00 | 2.00 |
| Liquid | DeepSeek | 3.00 | 4.00 |
| Liquid | Grok | 3.00 | 3.00 |
| Liquid | ChatGPT | 3.00 | 4.00 |
| Liquid | DeepSeek | 3.00 | 5.00 |
| Liquid | Grok | 3.00 | 4.00 |
| Liquid | ChatGPT | 2.00 | 3.00 |
| Liquid | DeepSeek | 4.00 | 4.00 |
| Liquid | Grok | 3.00 | 3.00 |
| Liquid | ChatGPT | 3.00 | 3.00 |
| Liquid | DeepSeek | 3.00 | 3.00 |
| Liquid | Grok | 3.00 | 2.00 |
| Liquid | ChatGPT | 2.00 | 2.00 |
| Liquid | DeepSeek | 3.00 | 3.00 |
| Liquid | Grok | .00 | 1.00 |
| Pureed | ChatGPT | 4.00 | 2.00 |
| Pureed | DeepSeek | 5.00 | 3.00 |
| Pureed | Grok | 6.00 | 3.00 |
| Pureed | ChatGPT | 6.00 | 6.00 |
| Pureed | DeepSeek | 6.00 | 5.00 |
| Pureed | Grok | 6.00 | 6.00 |
| Pureed | ChatGPT | 6.00 | 5.00 |
| Pureed | DeepSeek | 6.00 | 5.00 |
| Pureed | Grok | 6.00 | 6.00 |
| Pureed | ChatGPT | 5.00 | 5.00 |
| Pureed | DeepSeek | 5.00 | 6.00 |
| Pureed | Grok | 6.00 | 6.00 |
| Pureed | ChatGPT | 5.00 | 5.00 |
| Pureed | DeepSeek | 6.00 | 5.00 |
| Pureed | Grok | 6.00 | 6.00 |
| Pureed | ChatGPT | 6.00 | 5.00 |
| Pureed | DeepSeek | 6.00 | 6.00 |
| Pureed | Grok | 5.00 | 5.00 |
| Solid | ChatGPT | 6.00 | 5.00 |
| Solid | DeepSeek | 6.00 | 4.00 |

| | | | |
|---|---|---|---|
| Solid | Grok | 6.00 | 6.00 |
| Solid | ChatGPT | 4.00 | 4.00 |
| Solid | DeepSeek | 6.00 | 5.00 |
| Solid | Grok | 7.00 | 7.00 |
| Solid | ChatGPT | 5.00 | 5.00 |
| Solid | DeepSeek | 6.00 | 4.00 |
| Solid | Grok | 7.00 | 6.00 |
| Solid | ChatGPT | 6.00 | 6.00 |
| Solid | DeepSeek | 6.00 | 6.00 |
| Solid | Grok | 7.00 | 6.00 |
| Solid | ChatGPT | 5.00 | 4.00 |
| Solid | DeepSeek | 5.00 | 5.00 |
| Solid | Grok | 7.00 | 7.00 |
| Solid | ChatGPT | 6.00 | 5.00 |
| Solid | DeepSeek | 6.00 | 5.00 |
| Solid | Grok | 7.00 | 4.00 |

Supplementary Table S2. Inter-rater reliability for total guideline-compliance scores across all menus (N = 54)

| **Statistic** | **Value** | **95% Confidence Interval** | **Value** | **p-value** |
|---|---|---|---|---|
| ICC (Single Measures) | 0.776 | 0.637 – 0.866 | 8.473 | < 0.001 |
| ICC (Average Measures) | 0.874 | 0.778 – 0.928 | 8.473 | < 0.001 |
